# Step‐by‐step insight into gait analysis: A narrative review unlocking knee biomechanics

**DOI:** 10.1002/ksa.70067

**Published:** 2025-09-16

**Authors:** Giovanni Spallone, Letizia Mancini, Arianna Carnevale, Stefano Campi, Alessandro de Sire, Emiliano Schena, Pieter D'Hooghe, Michael T. Hirschmann, Rocco Papalia, Umile Giuseppe Longo

**Affiliations:** ^1^ Fondazione Policlinico Universitario Campus Bio‐Medico Rome Italy; ^2^ Research Unit of Measurements and Biomedical Instrumentation, Department of Engineering Università Campus Bio‐Medico di Roma Rome Italy; ^3^ Research Unit of Orthopaedic and Trauma Surgery, Department of Medicine and Surgery Università Campus Bio‐Medico di Roma Rome Italy; ^4^ Physical and Rehabilitative Medicine, Department of Medical and Surgical Sciences University of Catanzaro “Magna Graecia” Catanzaro Italy; ^5^ Department of Orthopaedic Surgery and Sports Medicine Aspetar Hospital Doha Qatar; ^6^ University Clinic for Orthopedic Surgery and Traumatology, Kantonsspital Baselland Bruderholz Schweiz; ^7^ Department of Clinical Research, Research Group Michael T. Hirschmann, Regenerative Medicine & Biomechanics University of Basel Basel Schweiz

**Keywords:** anterior cruciate ligament deficiency, kinematics, kinetics, knee osteoarthritis, optoelectronic motion capture systems

## Abstract

Gait analysis offers a powerful tool for clinical and orthopaedic decision‐making. By quantifying spatiotemporal, kinematic and kinetic parameters during walking, it provides a dynamic window into joint function that static imaging cannot capture. Despite its potential, gait analysis remains largely confined to specialised centres, with limited integration in clinical pathways, mainly due to its perceived complexity and lack of standardisation. This narrative review aims to bridge that gap through a step‐by‐step approach to guide orthopaedic surgeons, sports medicine physicians and musculoskeletal clinicians in understanding and interpreting key biomechanical markers relevant to common knee pathologies, such as osteoarthritis and anterior cruciate ligament injury. Particular attention is given to how deviations in parameters like joint angles and moments, dynamic alignment and centre of pressure trajectories can offer actionable insights into disease progression, treatment response and surgical planning. The urgent need for standardised protocols, encompassing marker placement, biomechanical modelling and data processing, is also underscored, as they are essential to ensure reproducibility and facilitate clinical translation. By clarifying the clinical meaning of gait metrics, this review empowers healthcare professionals to integrate dynamic functional data into everyday decision‐making and move towards more personalised, biomechanically informed care.

**Level of Evidence:** Level IV.

**Abbreviations**
ACL: anterior cruciate ligament
AP: antero‐posterior
BMI: body mass index
COP: centre of pressure
dHKAA: dynamic hip‐knee‐ankle angle
GRF: ground reaction force
HKAA: hip‐knee‐ankle angle
HS: heel strike
KAA: knee adduction angle
KAM: knee adduction moment
KFA: knee flexion angle
KFM: knee flexion moment
KIRM: knee internal rotation moment
KOA: knee osteoarthritis
LR: loading response
ROM: range of motion
STPs: spatio‐temporal parameters
TO: toe off
TS: terminal stance
vGRF: vertical ground reaction force

## INTRODUCTION

Gait analysis is the systematic study of human locomotion, primarily focused on quantifying joint function and movement quality through objective biomechanical measures [20, 67, 74, 150]. In clinical practice, gait analysis allows clinicians not only to characterise musculoskeletal and neurological diseases through the identification of gait abnormalities [10, 20, 24, 42, 43, 60, 67, 74, 89, 102, 147], but also to monitor functional recovery and performance following surgical procedures or conservative treatments [116, 147].

This evaluation can be performed using different methods, ranging from simple visual observation to advanced instrumented systems [4, 74, 145]. Among the available technologies, the most widely adopted and extensively validated approach combines optoelectronic motion capture systems with force plates [19, 43, 63, 74, 145, 148]. This setup enables a comprehensive assessment of how patients move, thus providing valuable insights to support clinical decision‐making [20, 74, 135, 136].

Despite the diagnostic and functional value of these data, the translation of gait analysis into clinical practice remains limited [147], mainly due to the complexity of the outputs and the lack of training among clinicians unfamiliar with biomechanical concepts [43]. To address this gap, the goal of the present work is to provide a step‐by‐step interpretive guide aimed at helping orthopaedic surgeons, sports medicine physicians and musculoskeletal clinicians better understand gait analysis results and apply them in clinical reasoning. By offering a clear and structured overview of the key biomechanical parameters, this narrative review seeks to support the integration of gait data into routine medical practice and enhance their relevance for diagnosis, treatment planning and outcome monitoring.

## THE GAIT CYCLE PHASES

A solid knowledge of the gait cycle is essential for interpreting motion data and linking movement patterns to underlying musculoskeletal function. Before exploring healthy and pathological patterns, it is important to review the typical phases of gait cycle.

**Figure 1:**
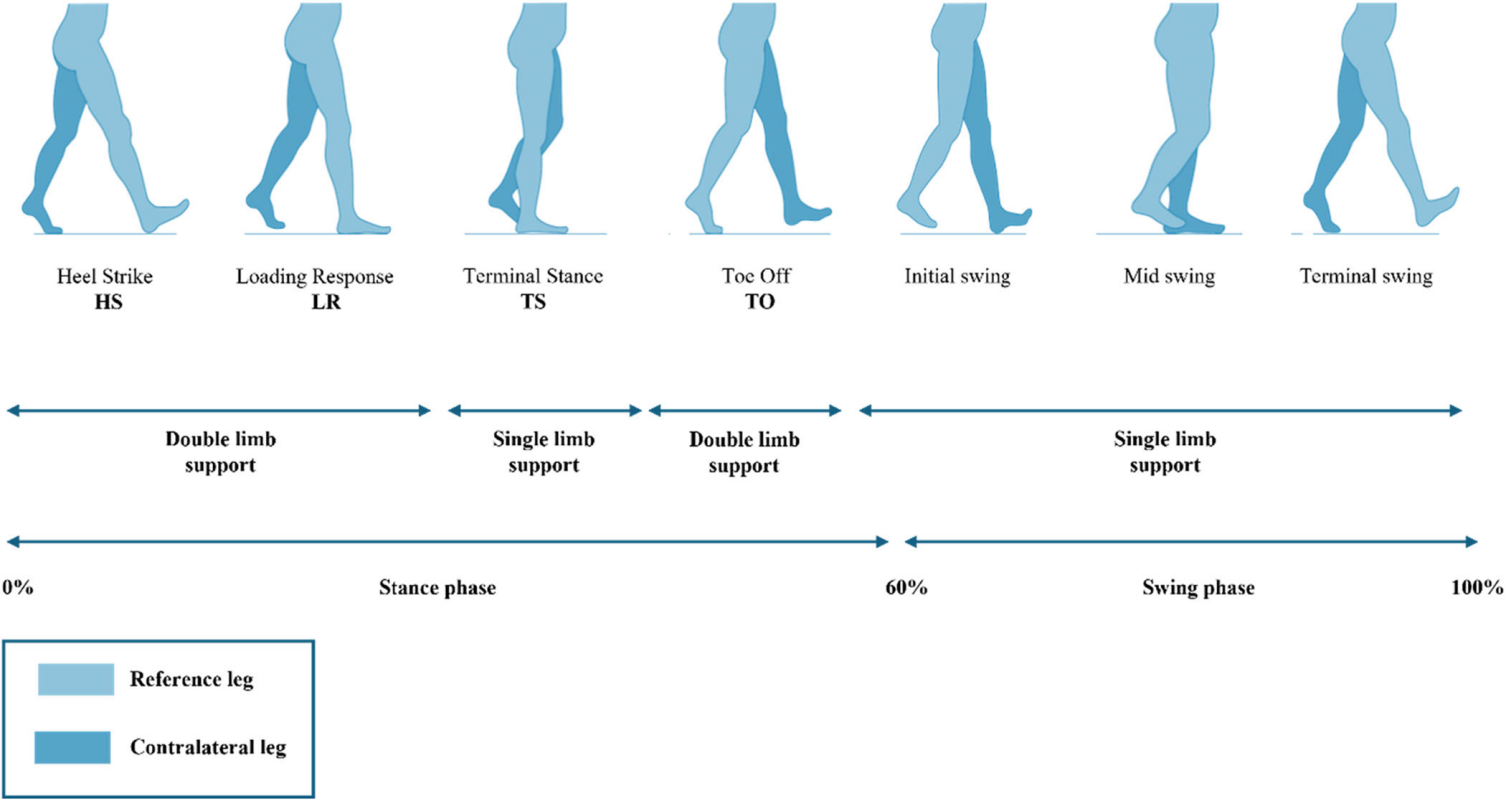
Representation of gait cycle phases. HS, heel strike; LR, loading response; TO, toe off; TS, terminal stance.

The gait cycle (Figure 1) refers to the sequence of movements performed by a lower limb during walking, beginning and ending with the same foot contacting the ground [20, 67, 74, 143]. It is traditionally divided into two main phases: the stance phase, during which the foot is in contact with the ground (approximately 60% of the gait cycle), and the swing phase, when the foot moves forward without ground contact (about 40% of the cycle) [20, 61, 67, 74, 143, 147]. Each of these phases can be further subdivided into specific subphases that reflect distinct biomechanical roles in locomotion:

- *Heel strike (HS) or initial contact*: it marks the beginning of the gait cycle, when the foot first contacts the ground (0%–2% of the gait cycle); [20, 67, 74, 143].
- *Loading response (LR) or foot flat*: it occurs as the reference leg accepts the body's full weight (2%–12% of the gait cycle); [20, 67, 74, 143].
- *Mid stance*: it is the midpoint of the stance phase (12%–30% of the gait cycle); [20, 67, 74, 143].
- *Terminal stance (TS) or heel off*: it initiates as the heel begins to lift off the ground (30%–50% of the gait cycle); [20, 67, 74, 143].
- *Toe off (TO) or preswing*: it occurs as the toe leaves the ground, marking the transition into the swing phase (50%–60% of the gait cycle); [20, 67, 74, 143].
- *Initial swing*: the foot accelerates forward (60%–73% of the gait cycle); [20, 67, 74, 143].
- *Mid‐swing*: it represents the midpoint of the swing phase, when the limb continues moving forward (73%–87% of the gait cycle); [20, 67, 74, 143].
- *Terminal swing*: the limb decelerates in preparation for the next HS (87%–100% of the gait cycle) [20, 67, 74, 143].

## KEY GAIT ANALYSIS PARAMETERS

The goal of clinical gait analysis is to provide quantitative data that characterise patient's gait biomechanics. These data are commonly grouped in four distinct categories: anthropometric, spatiotemporal, kinematic and kinetic [20, 74, 147]. Each category contributes to a different aspect of movement analysis and can support clinical interpretation in various ways.

*Anthropometric parameters* refer to patient‐specific characteristics such as weight, height, age, body mass index (BMI) [74]. A comprehensive consideration of these variables is crucial for accurate and reliable data interpretation [150].

*Spatiotemporal parameters* (STPs) describe time‐ and distance‐related measurements [20, 37, 60, 74, 122, 147]. The most used STPs are walking speed, cadence, step and stride lengths [20, 37, 60, 74, 122, 147]. These are among the easiest parameters to measure and have been associated with the level of disability and functional impairment in various disease conditions [60, 74]. Notably, STPs are known to depend on individual anthropometric characteristics, such as height and leg length. Although this affects intersubject comparability, values are presented without normalisation, consistent with standard reporting in the literature and clinical gait practice [12, 53, 122, 133]. Table 1 provides an overview of the commonly used STPs, including their definitions, measurement units, reference values and clinical relevance.

**Table 1:** Spatiotemporal parameters: definition, reference values, clinical relevance [14, 112].

Parameter	Definition	Typical values (adult)	Clinical relevance
Walking speed (m/s)	Distance walked per unit of time	1.18–1.35 (men) 1.11–1.28 (women)	Indicator of global mobility; predictor of fall risk and disability
Cadence (step/min)	Number of steps taken per minute	114–120 (men) 121–129 (women)	Altered in gait disorders and compensatory strategies
Step length (m)	Distance between two consecutive heel strikes of opposite feet	0.61–0.65 (men) 0.53–0.59 (women)	Reduced in elderly, pathological condition (as Parkinson disease, knee osteoarthritis, stroke) and immediate postoperative conditions
Stride length (m)	Distance between two consecutive heel strikes of the same foot	1.22–1.30 (men) 1.06–1.20 (women)	Reflects gait symmetry and efficiency

*Kinematic parameters* describe joint angles (knee flexion/extension angle, knee adduction/abduction angle, knee internal/external rotation angle, hip‐knee‐ankle angle [HKAA]), range of motion (ROM) and segment trajectories [20, 37, 60, 74, 84, 122, 124, 143, 147]. They are commonly represented through time‐normalised curves across the gait cycle [38, 44, 92, 147], providing valuable insight into movement quality and deviations from healthy joint patterns. A summary of the main kinematic parameters is provided in Table 2.

**Table 2:** Kinematic parameters during gait: definition, reference values, clinical relevance [24, 67, 96, 124, 143].

Parameter	Definition	Typical values range of motion (ROM)(adult)	Clinical relevance
Knee flexion/extension angle (°)	Angular displacement of the knee in the sagittal plane (flexion‐extension)	0°–60°	Reduced peak flexion is typical of stiff knee and limits foot clearance.
Knee adduction/abduction angle (°)	Angular deviation of the knee in the frontal plane (varus‐valgus)	0°–5°	Increased adduction linked to medial knee osteoarthritis and prosthetic overload, while excessive abduction is associated with frontal plane instability.
Knee internal/external rotation angle (°)	Rotation of the tibia relative to the femur in the transverse plane	10°–15°	Altered ROM is commonly associated with anterior cruciate ligament instability.
Dynamic hip‐knee‐ankle angle (°)	Frontal plane lower limb alignment during functional tasks	It depends on the patient anatomy	Marked varus/valgus deviations suggest structural malalignment; frontal plane sign shifts indicate knee instability.

*Kinetic parameters* reflect the internal and external loads acting on the body during walking. They include ground reaction forces (GRFs) [20, 37, 60, 74, 122, 143, 147], that is, forces exchanged between the foot and ground, and moments [20, 37, 60, 74, 122, 143, 147], which represent the rotational effect generated by forces around joints [144]. Joint Moments are typically classified into internal, primarily generated by muscle activity, and external, resulting from GRFs [20, 147]. These moments have the same magnitudes, but opposite signs [20, 147]. Additionally, the centre of pressure (COP) is a key kinetic parameter that defines the point of application of the resultant GRF and reflects how pressure is distributed over a supporting surface during the stance phase [105]. In clinical practice, these parameters are valuable tools for assessing compensatory strategies and identifying abnormal joint loading. Table 3 summarises the main kinetic variables relevant to knee biomechanics, including their definition and clinical implications.

**Table 3:** Kinetic parameters: definition, clinical relevance [67, 96, 124, 143].

Parameter	Definition	Clinical relevance
Ground reaction force [N]	Force exerted by the ground on the foot during stance phase	Detects interlimb loading asymmetries and is useful for monitoring postoperative recovery.
Knee flexion/extension moment (Nm/kg)	Moment that tends to flex/extend the knee (sagittal plane)	Reflects quadriceps load during weight acceptance; relevant for sagittal plane knee stability.
Knee adduction/abduction moment (Nm/kg)	Moment that tends to rotate the knee medially/laterally (frontal plane)	Reflects knee medial compartment load; often increased in knee osteoarthritis and relevant for prosthetic longevity.
Knee internal/external rotation moment (Nm/kg)	Moment that tends to rotate the knee internally/externally around the longitudinal axis	Reflects knee rotational loading; altered profiles are common in anterior cruciate ligament‐deficient knees.
Centre of pressure path (m)	Trajectory of the point of application of the total GRF under the foot	Indicates dynamic postural control; altered patterns reflect instability, asymmetries and compensatory mechanisms.

## DATA SOURCE AND BIOMECHANICAL PROCESSING

To ensure methodological transparency, the data source and processing pipeline are briefly described below.

This narrative review does not include any meta‐analysis or statistical pooling of data across studies. Instead, it offers a descriptive synthesis of key biomechanical concepts to support clinical interpretation of gait analysis. All quantitative gait curves shown in the figures are derived from our database and presented solely for illustrative purposes. In addition, no formal risk of bias or quality appraisal tools were applied, as the present narrative review does not involve systematic study selection or formal evidence synthesis.

Data were acquired using the Istituto Ortopedico Rizzoli (IOR) marker set [83] on an optoelectronic motion capture system (Qualisys, Sweden) with two integrated force plates (AMTI, USA). Joint angles were calculated using a Cardan XYZ rotation sequence [146], while joint moments were computed via inverse dynamics [100] and reported as net external moments, normalised to body weight. The signals shown in the figures represent the mean of five repeated gait trials for each subject and correspond to three groups: healthy adults (blue line), patients with unilateral knee osteoarthritis (KOA) of Kellgren–Lawrence grade ≥3 [72] (red line), and individuals with ACL deficiency (green line).

## GAIT ANALYSIS DATA: CLINICAL INTERPRETATION

Once gait parameters have been extracted, understanding their clinical meaning is essential to assess joint function and pathological adaptations. This section provides a practical overview of anthropometric, spatiotemporal, kinematic and kinetic variables.

### Anthropometric parameters

Among anthropometric parameters, BMI deserves particular attention, as it provides a useful estimate of a patient's weight status [150] and helps identify cases of overweight or obesity [127, 142]. According to the World Health Organization, a BMI between 18.5 and 24.9 kg/m² is considered within the normal weight range [127, 142], while values exceeding this threshold require careful interpretation. This consideration is particularly relevant in obese individuals (BMI ≥ 30 [127, 142]), where the identification of anatomical landmarks may be challenging due to excessive adipose tissue. Furthermore, soft tissue artefacts, caused by the relative movement of skin and subcutaneous tissues during dynamic activities, may compromise the accuracy of joint kinematics and kinetics estimation [82, 118].

### Spatiotemporal parameters

STPs provide essential information about the rhythm and coordination of gait and are often the first indicators of functional impairment [74]. Their interpretation can offer immediate clinical insights, especially for musculoskeletal or neurological diseases [42, 74, 104].

Notably, *walking speed* is one of the most informative indicators, as it reflects the overall functional capacity and energy efficiency of gait. A reduction in walking speed is commonly observed in patients with KOA, poststroke condition or Parkinson's disease, and has been associated with increased risk of falls and decreased independence [41, 60, 70, 81, 104, 107, 108, 125, 140, 152]. Values below 1.0 m/s are generally considered pathological and may indicate severe gait dysfunction [108].

*Cadence,* closely related to walking speed, also offers clinically relevant insights. It is often reduced in individuals with KOA and poststroke [10, 41, 42, 104, 108, 140]. Conversely, it may increase in Parkinson's patients as a compensatory strategy to maintain walking speed despite shortened step length [3, 106].

**Figure 2:**
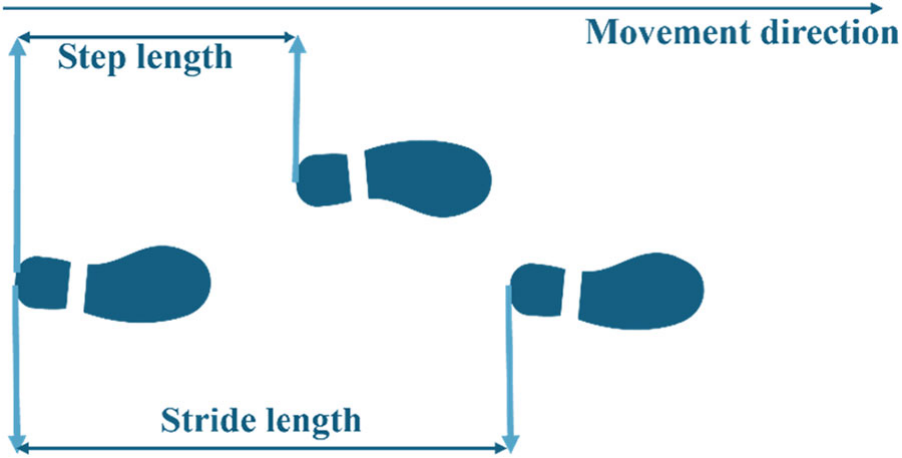
Stride and step length.

Finally, *step* and *stride length* (Figure 2) complete the picture of STPs. These parameters are closely related to limb advancement and gait symmetry. Reduced values, along with prolonged stride duration on one side, are typical asymmetrical findings in patients with unilateral knee pathologies [35, 42, 104, 108, 119, 139]. A symmetrical reduction in stride length, on the other hand, may indicate generalised deconditioning or underlying neuromuscular disorders [3, 107].

### Kinematic parameters

To better understand knee biomechanics during gait, it is essential to consider its 6 *degrees of freedom*, which define its complex motion across three anatomical planes: flexion and extension in the sagittal plane, varus and valgus stress (adduction and abduction) in the frontal plane, and internal and external rotation in the transverse plane [1, 24, 93, 101, 144, 153].

**Figure 3:**
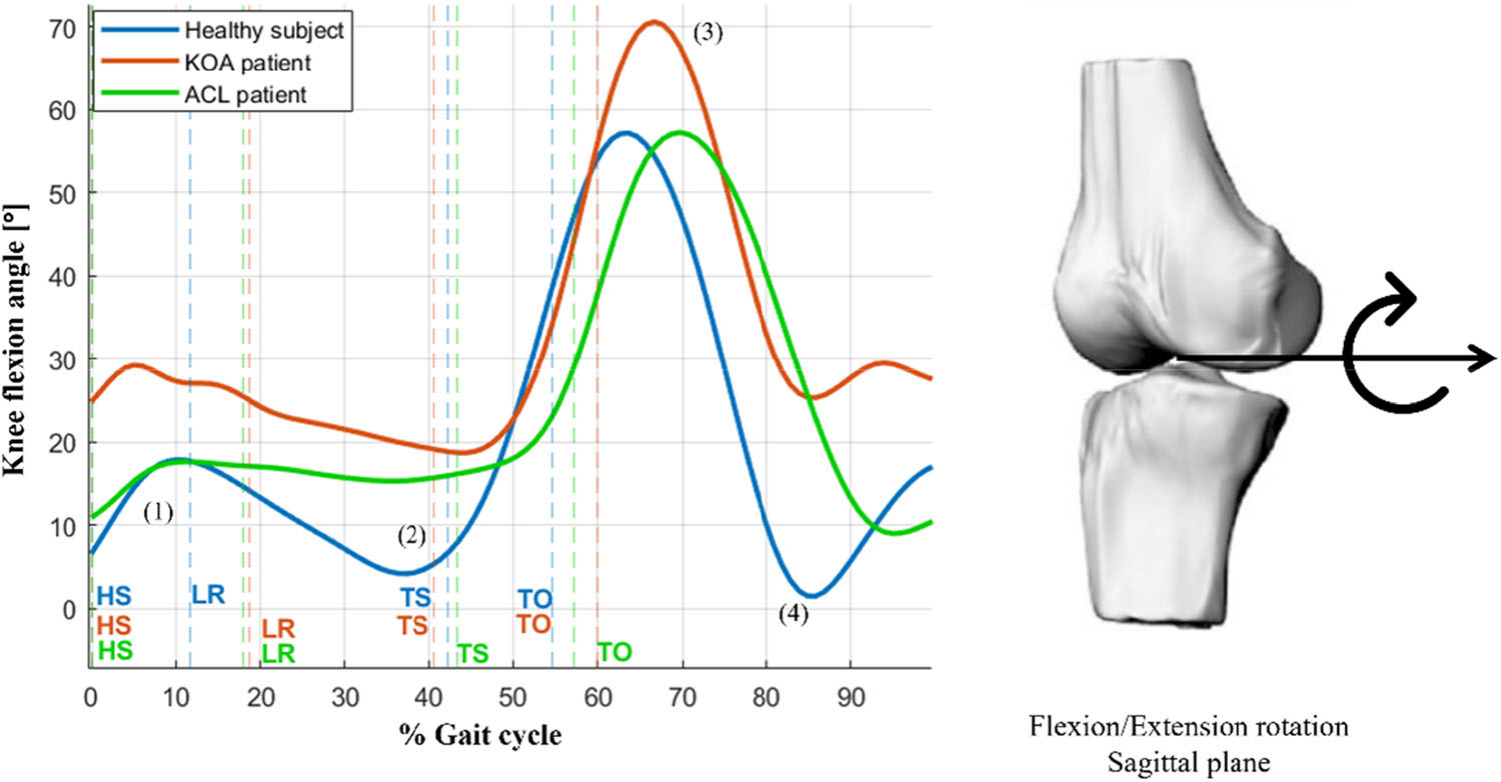
Mean knee flexion angle during the gait cycle for healthy adults (blue), patients with KOA (red) and patients with ACL deficiency (green). Data represent the mean of five gait trials per subject, normalised to the gait cycle (%). ACL, anterior cruciate ligament; HS, heel strike; KOA, knee osteoarthritis; LR, loading response; TO, toe off; TS, terminal stance.

Knee *flexion‐extension* (Figure 3) is the primary angular movement during gait [1, 24, 93, 96, 101, 144, 153]. In this type of kinematic plot, increasing values correspond to knee flexion, whereas decreasing values indicate extension. As illustrated in Figure 3, the knee exhibits two flexion (points 1 and 3) and two extension peaks (points 2 and 4) throughout the gait cycle. Points 1 and 2 occur during the stance phase, while points 3 and 4 are observed in the swing phase. The peak flexion angle in the swing phase (point 3) consistently exceeds that in the stance phase (point 1), with typical physiological values around 60° and 20°, respectively [24, 153]. Excessive deviations from these normative values may indicate underlying gait abnormalities and should prompt further clinical investigation to identify potential musculoskeletal or neuromotor impairments.

The overall shape of the knee flexion angle (KFA) curve is similar between healthy subject (blue line) and KOA patients (red line), but the ROM is generally lower for the osteoarthritic knees [139, 153]. Clinically, this suggests that while the motor pattern is preserved, the reduced ROM may reflect joint stiffness or pain, highlighting the need for targeted interventions aimed at restoring mobility [24, 153]. As shown in Figure 3, during early stance, the healthy knee reaches a flexion peak of approximately 15°–20°, allowing for effective shock absorption during weight acceptance [24, 153]. In contrast, the KOA patient (red line) displays a higher flexion angle at HS and a more pronounced early stance peak [70, 153], likely reflecting a compensatory strategy. As the gait progresses into mid‐stance, the healthy knee extends towards full extension, promoting postural stability and efficient forward progression. Conversely, the KOA patient maintains a consistently flexed knee posture, suggesting impaired extension capacity, a common feature of KOA [24, 70, 90, 153]. This altered pattern persists in the swing phase: while the healthy knee reaches its second flexion peak before returning to extension in preparation for the next HS, the osteoarthritic one continues to exhibit a flexed alignment, suggesting persistent joint stiffness or compensatory motor patterns [24, 153]. Recognising this deviation is crucial, as it may indicate the need for targeted rehabilitative strategies aimed at restoring knee extension capacity. Additionally, in more advanced stages, the persistence of a flexed posture might support the indication for surgical intervention to address fixed flexion deformities and improve functional outcomes [85, 134].

Regarding ACL patients (green line), KFA pattern generally follows the same biphasic shape seen in healthy individuals. However, differences emerge in the second half of the stance phase, where ACL patients tend to maintain a more flexed knee posture, reflecting residual instability or cautious weight acceptance [16, 17, 36, 55]. Furthermore, the overall ROM appears slightly reduced suggesting neuromuscular or proprioceptive deficits [17, 132, 153], and protective mechanisms to reduce anterior tibial translation during dynamic loading [16, 55, 153]. This altered pattern might help identify patients who are at increased risk of functional failure under conservative management [75]. Clinical gait analysis may therefore contribute to decision‐making regarding surgical reconstruction, particularly in individuals who fail to regain adequate joint control during demanding tasks.

**Figure 4:**
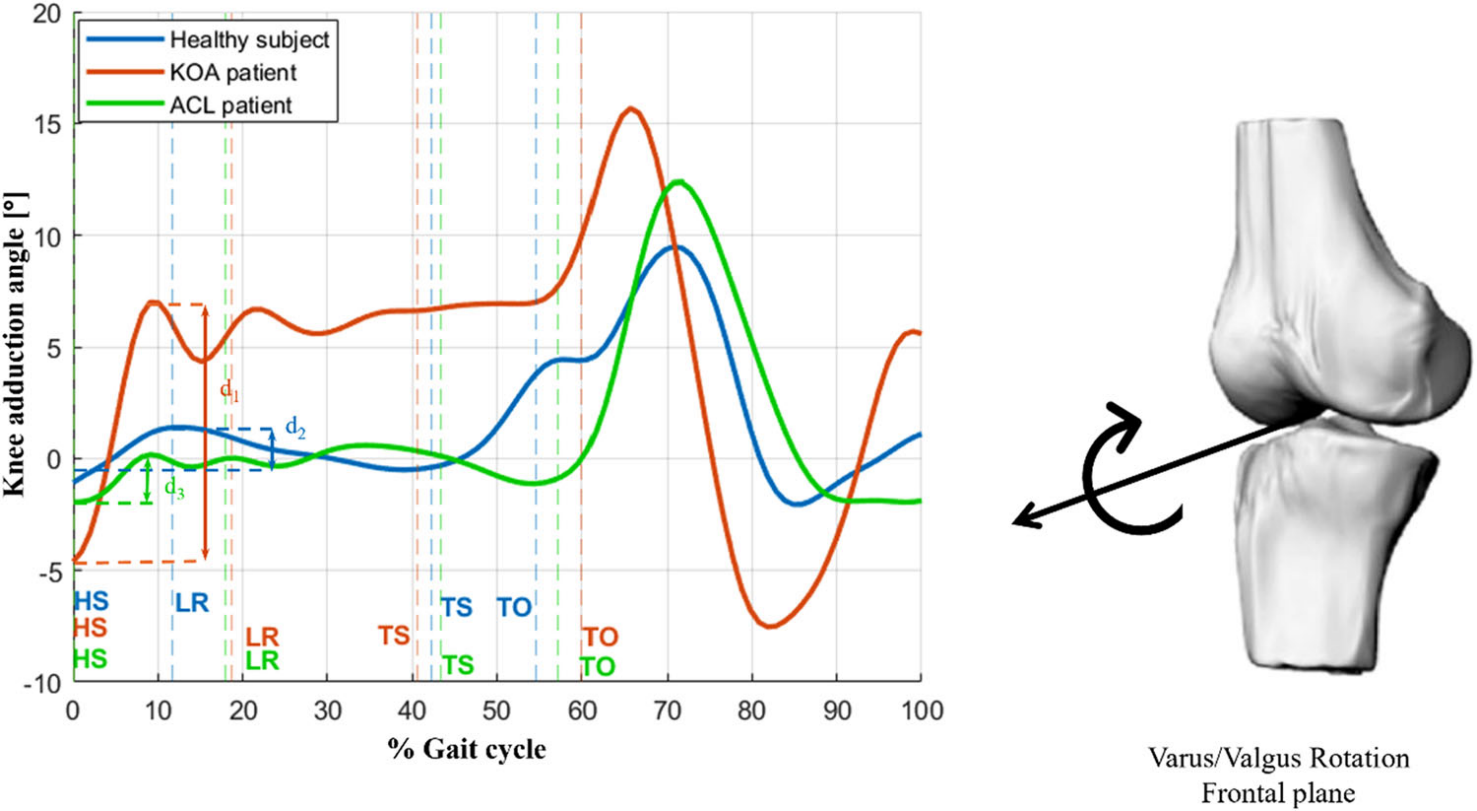
Mean knee adduction angle during the gait cycle for healthy adults (blue), patients with KOA (red) and patients with ACL deficiency (green). Data represent the mean of five gait trials per subject, normalised to the gait cycle (%). ACL, anterior cruciate ligament; HS, heel strike; KOA, knee osteoarthritis; LR, loading response; TO, toe off; TS, terminal stance.

Regarding frontal plane kinematics, *knee adduction angle* (KAA) quantifies the angular displacement of the tibia relative to the femur along the medio‐lateral axis [24, 39, 144] (Figure 4). In healthy individuals, KAA remains close to zero throughout the stance phase (Figure 4, blue line) [74, 153]. The knee initially moves into slight abduction during early stance, gradually transitions into adduction as the gait cycle progresses through swing phase [24, 94], and returns to abduction towards the end of the cycle [24].

In contrast, KOA patients (red line) exhibit a larger range of KAA values [39, 74, 153]. Although the general shape is similar to the healthy subjects, the amplitude is markedly greater. Particularly, individuals with KOA tended to move into greater abduction during the loaded (stance) phase and into higher adduction during the unloaded (swing) phase. From a clinical standpoint, this exaggerated frontal plane excursion may reflect coronal plane instability and is often associated with varus deformity progression and asymmetric joint loading, contributing to medial compartment degeneration [39, 40].

Moreover, as shown in Figure 4, ACL patients (green line) generally exhibit a KAA profile similar in shape to healthy subjects, but with higher peak values during both stance and swing phases [16, 32, 55, 132, 153]. Although the magnitude is lower than in KOA patients, the variability and amplitude of KAA suggest an altered control of frontal plane knee motion [132]. Since increased KAA during dynamic loading has been associated with elevated ACL strain and higher risk of injury [9], monitoring these deviations may offer indirect yet clinically meaningful insight into ACL loading patterns, and assist in defining thresholds for neuromuscular control during functional activities.

Closely related to the KAA, the *varus thrust* refers to a rapid increase in varus alignment during the weight‐bearing phase of walking [21, 77, 94, 95]. It is typically quantified as the difference between the KAA at HS and its first maximum during the stance phase of gait (*d_i_* in Figure 4) [21, 39, 77, 88, 94, 95]. As illustrated in Figure 4, KOA patients show higher values of varus thrust compared to the healthy group (*d*_1_ > *d*_2_) [77, 94]. Both elevated KAA and varus thrust have been recognised as mechanical markers for disease severity and are considered key factors in osteoarthritis progression [39, 80, 129]. Clinically, a pronounced varus thrust might serve as a useful noninvasive marker to identify patients at increased risk of structural worsening [39, 80, 129], supporting the early adoption of preventive or conservative interventions such as bracing or gait retraining [46, 86].

On the other hand, in the context of ACL injury and reconstruction, varus thrust (*d*_3_) has also emerged as a clinically significant biomechanical factor. It is suspected to be a major contributor to the failure of ACL reconstructions [68]. Additionally, a direct relationship has been documented between varus thrust and increased tension on the ACL or its graft, especially during weight‐bearing activities. This excessive tensile load may lead to graft overstress, progressive instability and degenerative changes in the knee medial compartment [68, 73, 111]. For these reasons, clinical assessment and longitudinal monitoring are essential to identify patients at risk of graft failure and to develop targeted protective rehabilitation strategies.

**Figure 5:**
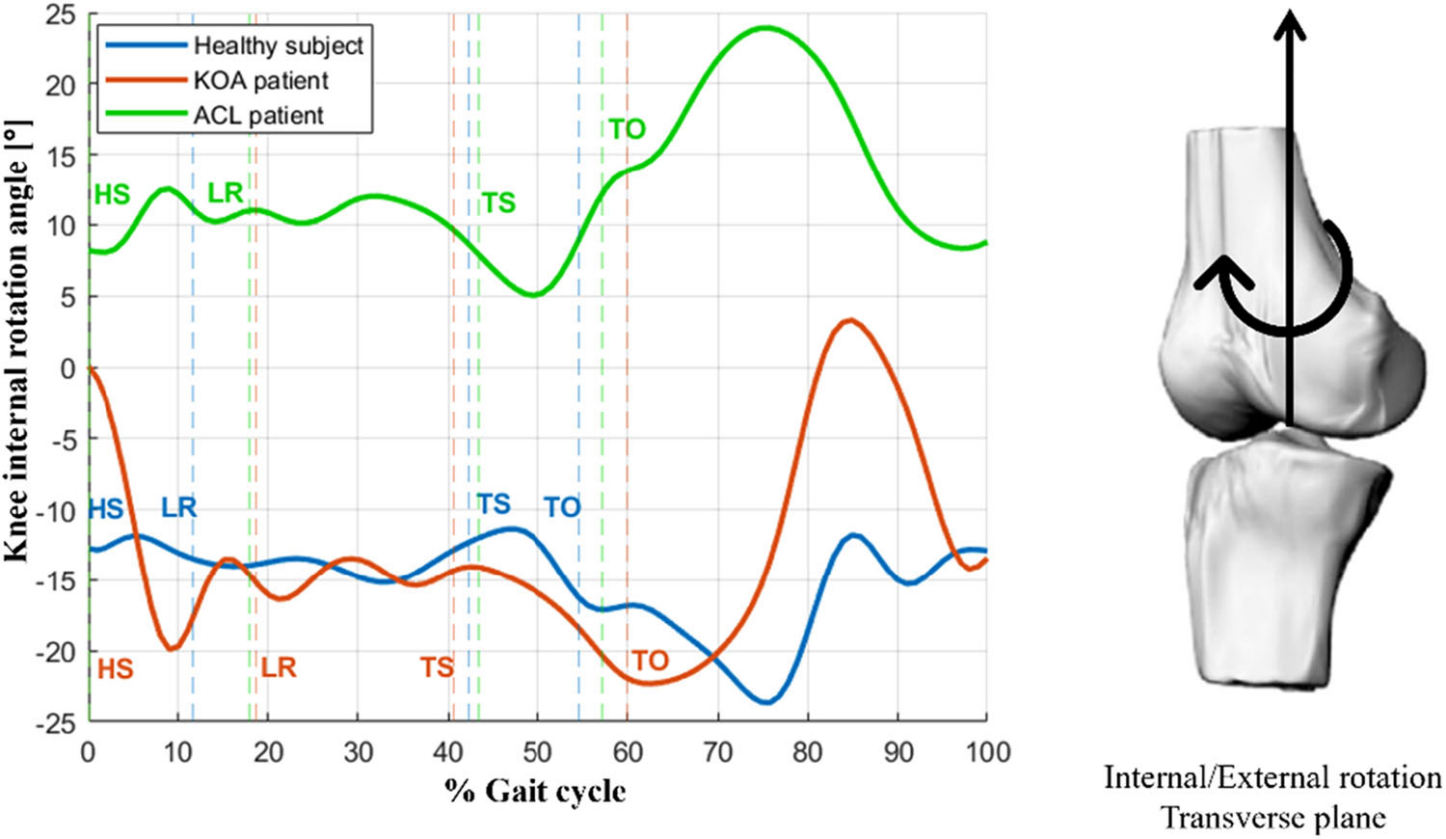
Mean knee internal rotation angle during the gait cycle for healthy adults (blue), patients with KOA (red) and patients with ACL deficiency (green). Data represent the mean of five gait trials per subject, normalised to the gait cycle (%). ACL, anterior cruciate ligament; HS, heel strike; KOA, knee osteoarthritis; LR, loading response; TO, toe off; TS, terminal stance.

In the context of transverse plane movements, knee joint motion involves *internal* and *external rotation* of the tibia relative to the femur (Figure 5) [24, 144, 147, 153]. These rotational movements are typically coupled with knee flexion and extension, as part of the so‐called screw‐home mechanism [24, 26, 101, 147, 153], as in early flexion the tibia undergoes internal rotation, whereas terminal extension is accompanied by external rotation, contributing to joint stability and efficient load transfer during gait [24, 26, 101, 147, 153]. Particularly, the healthy knee commonly exhibits internal‐external rotational excursions of approximately 5° [24]. As shown in Figure 5, at the beginning of the stance phase, the tibia is slightly externally rotated. Then both femur and tibia undergo internal rotation [24, 26, 101], and after the mid‐stance phase, they progressively rotate externally continuing this motion until the end of the gait cycle [24, 26, 101].

When considering KOA patients (red line), marked differences in internal rotation angle are primarily observed in the early stance phase [155]. As clear in Figure 5, following the other stance phases, there is a limitation in knee internal rotation, which is likely related to cartilage thinning [113]. Its clinical assessment could assist in both the diagnostic stratification of KOA severity and the tailoring of conservative or surgical interventions aimed at restoring joint mobility and optimising load distribution.

Notably, internal/external rotation is tightly regulated by the ACL, one of the primary knee stabilisers [24, 96, 101, 147]. It specifically ensures rotational stability and alignment during dynamic activities [24, 96, 101, 147]. When the ACL is injured or deficient, this stabilising function is compromised, often resulting in altered rotational kinematics, as depicted in Figure 5. To date, the interpretation of knee internal\external rotation patterns in ACL patients remains widely debated in the literature, due to the considerable variability observed across individuals [55, 141]. While some patients exhibit persistent external tibial rotation, others show an internal rotation bias, as illustrated in Figure 5. Despite these differences, both profiles deviate from the physiological pattern observed in healthy subjects suggesting a disruption of the normal screw‐home mechanism and a loss of internal rotational restraint. In a clinical perspective, an accurate assessment of knee rotational instability and laxity is essential for understanding the biomechanical consequences of ACL deficiency and evaluating the effectiveness of different reconstruction techniques aimed at restoring physiological joint function [64].

**Figure 6:**
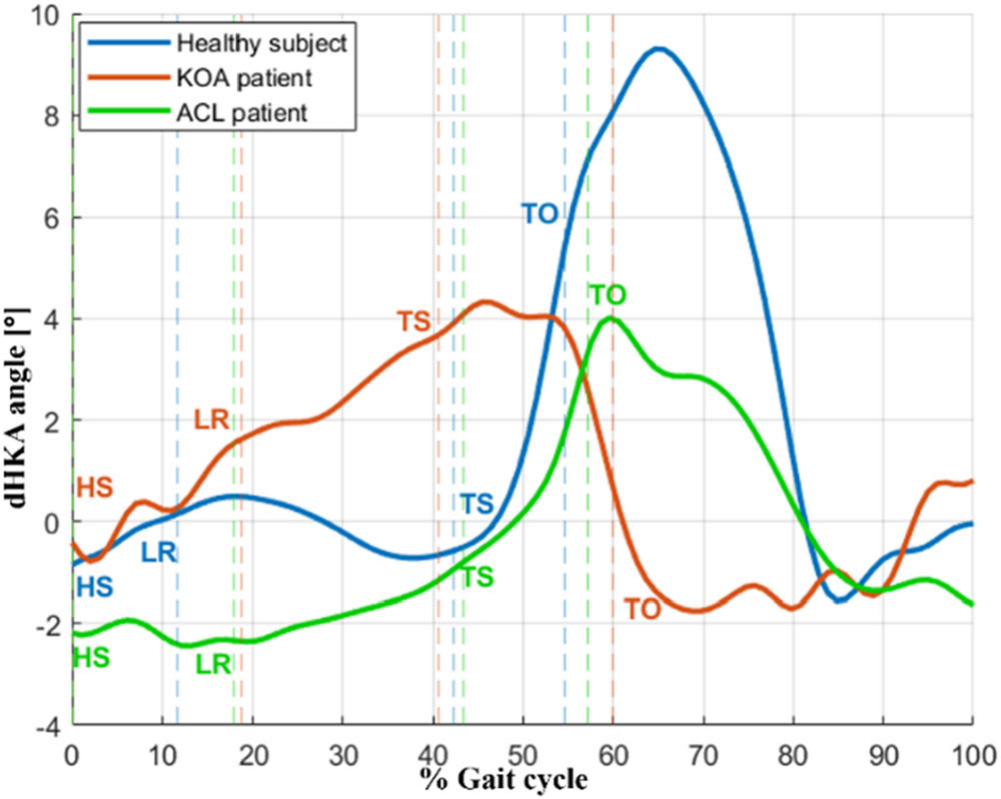
Mean dynamic hip‐knee‐ankle angle during the gait cycle for healthy adults (blue), patients with KOA (red) and patients with ACL deficiency (green). Data represent the mean of five gait trials per subject, normalised to the gait cycle (%). ACL, anterior cruciate ligament; HS, heel strike; KOA, knee osteoarthritis; LR, loading response; TO, toe off; TS, terminal stance.

Due to the growing recognition of its clinical relevance [27, 56, 65, 123, 154], the *dynamic hip‐knee‐ankle angle (dHKAA)* deserves specific attention among kinematic parameters. It describes the frontal plane lower limb alignment during gait [27, 56, 65, 123, 154]. It is computed by projecting the hip, knee, and ankle joint centres onto the coronal plane throughout the gait cycle [27, 65], in accordance with orthopaedic standards [28, 52]. To enable comparison with clinical radiographic assessments, 180° (neutral alignment) is set as the reference (zero) value. Accordingly, positive angles are classified as varus, while negative angles are classified as valgus (Figure 6). In this context, knees are typically classified as ‘stable’ if the dHKAA remains positive or negative, that is, in varus or valgus, for more than 95% of the stance phase [27]. Conversely, knees that change alignment classification during stance are defined as ‘changers’ [27]. As illustrated in Figure 6, frontal plane alignment is not a fixed parameter but instead varies in response to knee joint loading. An increase in positive dHKAA values reflects a shift towards greater varus alignment, while more negative values indicate a progression towards valgus. This dynamic behaviour highlights the importance of capturing joint alignment in motion, as static assessments may fail to reveal clinically relevant deviations [27, 56, 123]. As shown in Figure 6, healthy subjects exhibit a dynamic and smooth dHKAA pattern throughout the gait cycle, with a clear increase in varus alignment during mid‐stance followed by a return towards neutral configuration in late stance and swing. This behaviour reflects normal frontal plane control and adaptive joint loading in response to movement demands [27].

In contrast, KOA patient (red line) displays a markedly attenuated dHKAA curve, with persistently varus‐oriented alignment even during the early stance phase, when joint loading is at its peak. This early and sustained varus configuration leads to increased compressive forces on the medial compartment, contributing to cartilage degeneration and disease progression [39, 41, 80, 129, 139].

Frontal plane alignment can also be altered in ACL‐deficient patients, although the deviations are often smaller and more variable than those observed in KOA. As shown in Figure 6, ACL patients (green line) may exhibit a less pronounced varus excursion during mid‐stance and minor oscillations around the neutral axis throughout the stance phase. Unlike the ‘stable’ pattern seen in healthy subjects, ACL profile is indicative of a ‘changer’ behaviour [27], reflecting intermittent shifts between varus and valgus alignment and coronal plane instability. Clinically, the dynamic assessment of frontal plane alignment is particularly relevant, as ACL injuries are often associated with noncontact mechanisms involving excessive valgus or varus opening of the knee [22]. In this regard, abnormal coronal plane motion may therefore serve as both a marker of injury risk and a target for preventive or corrective interventions.

Finally, it is important to highlight that joint kinematics do not only provide descriptive information about movement patterns, but also play a critical role in the calculation of kinetic parameters. The estimation of joint moments relies on a tight interplay between segmental kinematics, joint centres of rotation and external forces [18]. Specifically, the orientation and relative motion of body segments, the definition of joint centres and rotation axes, and the spatial relation between these elements and the GRF vector all determine the moment arms through which forces act on the joints [18, 115]. Therefore, even small variations in joint angles or in the estimated location of anatomical reference points can substantially influence the magnitude and direction of the net external moments [18, 115]. This close interaction between motion and loading mechanics reinforces the need to interpret gait data globally, combining kinematic and kinetic insights to guide clinical decision‐making.

### Kinetic parameters

While kinematic data describe how a joint moves in space, kinetic parameters reveal why it moves that way [51, 124, 150]. They provide critical insight into the internal and external forces acting on the body during gait and are essential to understanding the mechanical demands placed on joints and muscles.

Among kinetic measures, *GRFs* represent the starting point, as they form the basis for computing all subsequent kinetic variables [147]. The interaction between foot and ground generates a three‐dimensional vector with vertical, anterior/posterior and medial/lateral components [51], however, in both clinical practice and research, the vertical component is most commonly analysed [51, 149]. Moreover, when the foot is not in contact with the ground, no GRF is generated, and all associated kinetic variables are effectively zero [96, 124].

**Figure 7:**
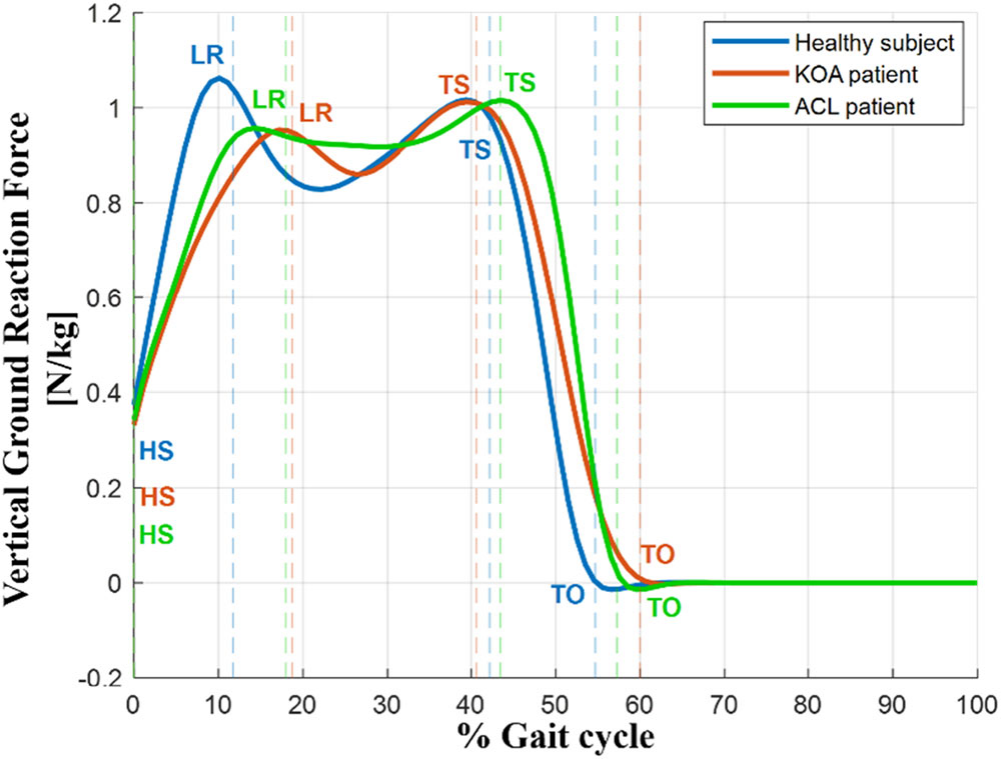
Mean vertical ground reaction force during the gait cycle for healthy adults (blue), patients with KOA (red) and patients with ACL deficiency (green). Data represent the mean of five gait trials per subject, normalised to the gait cycle (%). ACL, anterior cruciate ligament; HS, heel strike; KOA, knee osteoarthritis; LR, loading response; TO, toe off; TS, terminal stance.

As shown in Figure 7, the *vertical GRF* (vGRF) curve during walking exhibits a characteristic double‐peaked pattern, commonly referred to as the ‘M‐curve’ due to its resemblance to the shape of the letter [99]. In healthy individuals (blue line), this pattern reflects the typical progression of the stance phase. The first peak occurs shortly after HS, as body weight is rapidly transferred onto the limb and the downward velocity of the centre of mass is decelerated resulting in a vGRF that temporarily exceeds body weight. During midstance, the knee flexes and the force plate is partially unloaded, leading to a drop in vGRF below body weight. The second peak arises during TS, when the plantar‐flexor muscles are active and generate upward acceleration of the centre of mass, once again increasing vGRF values above body weight. Finally, it returns to zero as the contralateral limb takes over the support role and the foot leaves the ground [37, 71, 96, 99].

Individuals with KOA exhibit reduced peak vGRF and a less dynamic loading profile during gait. This pattern is clearly illustrated in Figure 7, where the vGRF curve (red line) shows lower overall peak loading and a blunted double‐hump profile compared to the healthy subject (blue line), especially during the LR and TS phases [13, 25, 91, 128]. From a biomechanical standpoint, articular cartilage behaves as a viscoelastic tissue, responding to mechanical loading in a time‐dependent manner [7]. A less dynamic loading pattern, as observed in KOA patients, may lead to prolonged and uneven cartilage deformation, increasing mechanical strain and potentially accelerating degenerative and inflammatory processes within the joint [13].

Furthermore, patients with ACL lesions also display characteristic alterations in vGRF profile [13, 34, 147]. These changes are often interpreted as compensatory strategies to protect knee joint or reflect neuromuscular deficits. As illustrated in Figure 7, ACL patients (green line) exhibit a markedly reduced and flattened vGRF pattern, with lower first and second peaks and a higher force plateau during midstance [13, 34]. This results in a less dynamic loading profile, characterised by sustained rather than cyclic limb loading throughout stance. Notably, an increased vGRF during midstance has been strongly associated with adverse changes in articular cartilage composition following ACL reconstruction [13]. This altered loading environment may contribute to early joint degeneration, highlighting the importance of restoring physiological gait mechanics. From a clinical perspective, the assessment of vGRF patterns during walking may serve as a valuable functional marker to detect abnormal joint loading and guide individualised rehabilitation strategies and treatments aimed at reducing cartilage stress and promoting long‐term joint preservation [13].

Following with the kinetic parameters' discussion, joint moments are commonly expressed in Nm/kg, as they are normalised to body weight to allow for comparison between individuals of different sizes. Rather than measuring individual muscle forces, joint moments give a global picture of how the joint is loaded and stabilised during gait. In the context of knee biomechanics, three moments must be discussed: knee flexion moment (KFM), knee adduction moment (KAM) and knee internal rotation moment (KIRM).

**Figure 8:**
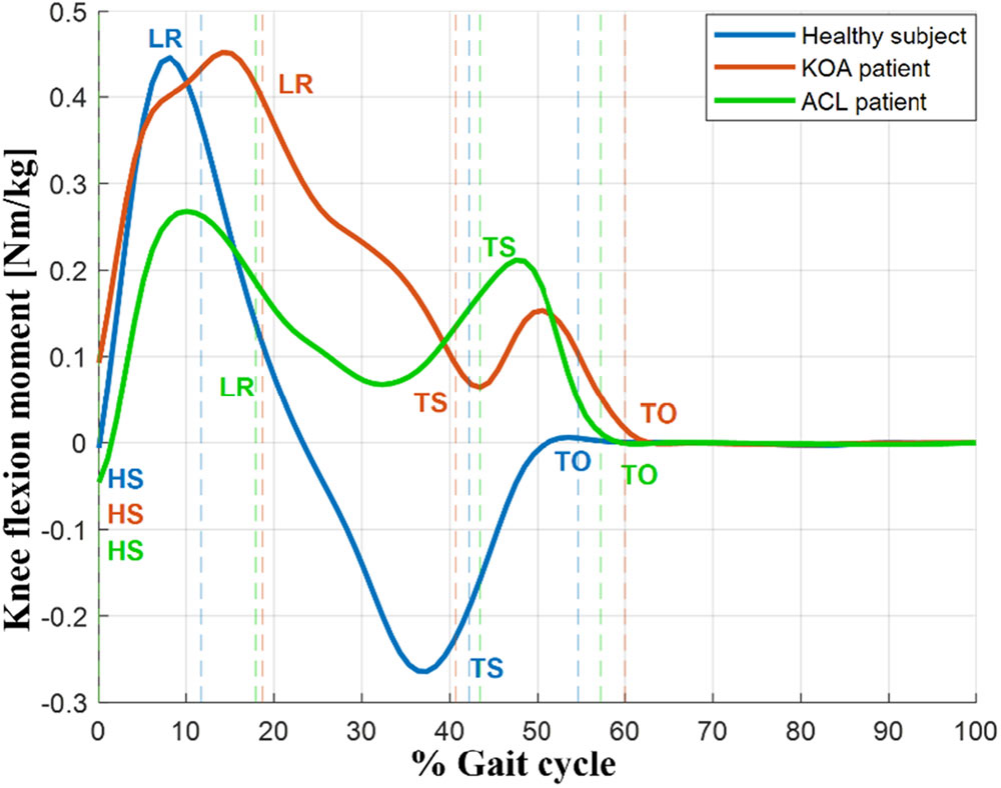
Mean knee flexion moment during the gait cycle for healthy adults (blue), patients with KOA (red) and patients with ACL deficiency (green). Data represent the mean of five gait trials per subject, normalised to the gait cycle (%). ACL, anterior cruciate ligament; HS, heel strike; KOA, knee osteoarthritis; LR, loading response; TO, toe off; TS, terminal stance.

*KFM* is the external moment that tends to flex the knee during the stance phase and it is primarily counteracted by the quadriceps [31]. As illustrated in Figure 8, the KFM typically reaches its peak during the first half of the stance phase, when the body's centre of mass moves forward over the supporting foot. In healthy individuals (blue line), the KFM shows a distinct peak in early stance, corresponding to weight acceptance, where the quadriceps eccentrically control knee flexion to stabilise the limb and attenuate impact forces. A second, smaller peak trough often occurs later in stance, reflecting a transient external extension moment balanced by internal knee flexor activity. This biphasic pattern represents an efficient control strategy to accommodate loading and propulsion during walking.

Patients with ACL lesion (green line) often exhibit reduced external KFMs during stance, a pattern known as quadriceps avoidance gait [58, 117]. This adaptation, first described by Berchuck et al. [11], is thought to minimise anterior tibial translation by limiting quadriceps activation [58, 147]. Importantly, this compensatory pattern often persists even after surgical reconstruction, highlighting the neuromuscular adaptations and the risk of early‐onset osteoarthritis [58]. In this context, gait analysis plays a pivotal role in objectively identifying such altered motor strategies, allowing clinicians to tailor rehabilitation programs, monitor recovery and adopt preventive measures.

However, altered sagittal plane knee kinetics are not limited to ACL‐injured patients. A similar pattern is consistently observed in individuals with KOA (red line), where reduced knee extension moments during stance [8, 33, 70, 153] characterise what has been defined as a ‘stiffened‐knee’ gait [33]. By altering the magnitude and distribution of contact forces within the tibiofemoral joint, these biomechanical changes may increase focal cartilage stress, trigger maladaptive remodelling responses, and ultimately contribute to the acceleration of osteoarthritic disease progression [5, 23, 33]. From a clinical perspective, early recognition and full understanding of stiff‐knee gait dynamics are essential to optimise patient care. Timely identification of this pattern can guide the selection of the most appropriate therapeutic approach, whether through targeted physical therapy or surgical intervention, to mitigate joint loading abnormalities and preserve long‐term joint function [48].

**Figure 9:**
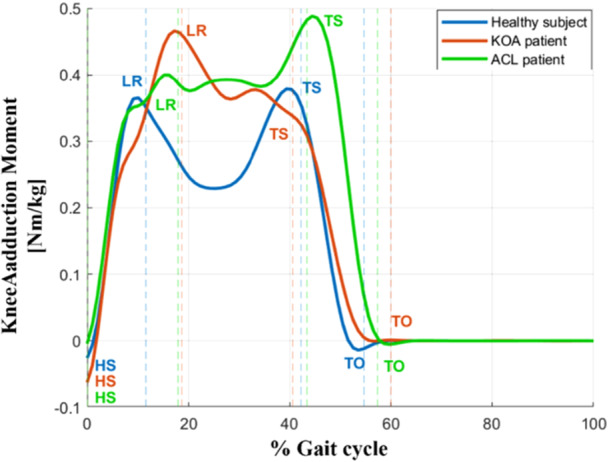
Mean knee adduction moment during the gait cycle for healthy adults (blue), patients with KOA (red) and patients with ACL deficiency (green). Data represent the mean of five gait trials per subject, normalised to the gait cycle (%). ACL, anterior cruciate ligament; HS, heel strike; KOA, knee osteoarthritis; LR, loading response; TO, toe off; TS, terminal stance.

*KAM* is a key kinetic variable and is commonly considered an index of medial knee joint loading [2, 24, 39, 40, 49, 131]. KAM represents the moment generated by the GRF relative to the knee joint centre in the frontal plane, tending to move the knee joint into adduction. In other words, KAM reflects how much GRF tends to rotate knee joint into varus alignment [49, 125]. Conversely, an abduction moment, i.e. when KAM became negative, would act to rotate the knee into a valgus alignment, thereby unloading the medial compartment and increasing the load on the lateral side [125]. In healthy individuals (blue line), the KAM typically exhibits a characteristic double‐peak pattern [124] during the stance phase (Figure 9). The first peak occurs during early stance (LR), as the body weight is transferred onto the limb. The second peak, generally slightly lower, appears in late stance (TS), as the centre of mass moves forward over the foot. Between these two peaks, a relative trough is observed during mid‐stance, reflecting the redistribution of forces across the joint.

KAM is particularly relevant in studies on KOA, as higher values have been consistently associated with increased medial compartment loading and potential disease progression [2, 39, 40, 49, 124, 131, 153]. A higher KAM implies a greater proportion of load transmitted through the medial compartment of the tibiofemoral joint, which is clinically relevant given that medial compartment overload is a known contributor to the development and progression of KOA [40, 49, 153]. Patients with KOA (red line) typically present with increased KAM values throughout the stance phase (Figure 9) [2, 39, 40, 49, 124, 131, 153]. Among these, the first peak of the KAM curve is of clinical interest, as it is often higher in magnitude and temporally shifted, reflecting both increased and prolonged knee medial compartment loading [24]. The second KAM peak, by contrast, is frequently reduced or even absent, a pattern likely linked to decreased push‐off force and altered propulsion mechanics [124]. Given its strong relationship with joint loading patterns, disease severity and progression, KAM also emerges as a crucial therapeutic target for both conservative and surgical strategies [24, 30, 45, 47].

In addition to its relevance in osteoarthritis, KAM is also altered in patients with ACL injury. However, findings across the literature remain inconsistent, reflecting the multifactorial nature of joint loading adaptations following ACL rupture [153]. Some studies report increased KAM peaks [54, 153], potentially due to compensatory frontal plane stiffness or neuromuscular control deficits. Others describe reduced or unchanged KAM values [132, 137, 153], possibly reflecting protective unloading strategies or interindividual variability in motor adaptation patterns. Despite these discrepancies in magnitude, a consistent observation is that the shape and timing of the KAM curve in ACL‐injured patients differ markedly from physiological patterns. As illustrated in Figure 9, ACL‐injured patients (green line) show a distinctly altered KAM profile compared to healthy controls (blue line), often characterised by reduced peak magnitude or temporally shifted peaks, suggesting deviations in medial‐lateral joint loading throughout stance phase. Therefore, even in the absence of clear consensus on peak values, the disruption of the physiological KAM pattern itself represents a clinically relevant marker of abnormal gait mechanics, with implications for rehabilitation, re‐injury prevention and early osteoarthritis development [76].

For clinicians, understanding the implications of the KAM curve is critical for interpreting gait analysis in KOA and ACL patients. It offers insight into joint loading patterns that may not be visible through kinematic parameters alone and provides a biomechanical rationale for therapeutic strategies tailored to offload the medial compartment and potentially delay disease progression [30, 45, 47, 76].

*KIRM* is a transverse plane kinetic parameter that plays a crucial role in controlling tibial rotation, ensuring stability and proper lower limb alignment.

**Figure 10:**
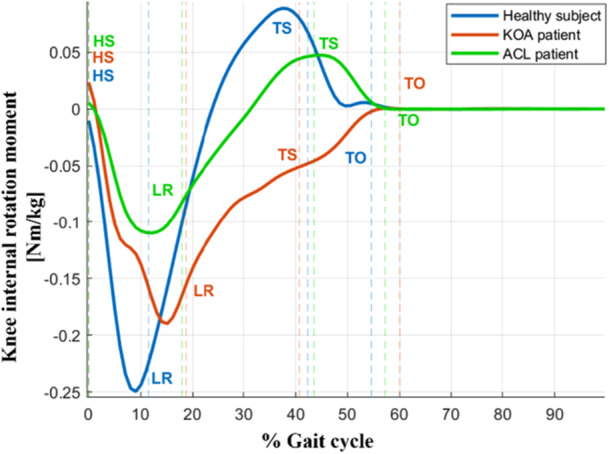
Mean knee internal rotation moment during the gait cycle for healthy adults (blue), patients with KOA (red) and patients with ACL deficiency (green). Data represent the mean of five gait trials per subject, normalised to the gait cycle (%). ACL, anterior cruciate ligament; HS, heel strike; KOA, knee osteoarthritis; LR, loading response; TO, toe off; TS, terminal stance.

Patients with KOA (red line) exhibit altered transverse plane loading patterns with reduced amplitudes compared to healthy subjects [124] (Figure 10). This tendency is clear in the early stance, as indicated by the attenuated negative deflection of the internal rotation moment curve [59, 78]. Such alterations likely reflect increased joint stiffness and reduced tibiofemoral rotational mobility [59, 78]. Moreover, KIRM has been shown to correlate with clinical symptoms, highlighting its use as sensitive biomechanical markers to monitor disease impact and guide therapeutic decision‐making [69].

Beyond its role in osteoarthritis, the KIRM is gaining attention as a surrogate measure of ACL load during dynamic tasks [57]. Specifically, KIRM is associated with elevated ACL injury risk [66] and is particularly relevant in the context of ACL injury mechanisms, as it contributes to the complex multiplanar loading environment that can compromise ligament integrity. Notably, the ACL is especially vulnerable when the knee is exposed to internal rotation moment at low flexion angles position. In these scenarios, the KIRM causes the tibia to rotate inward relative to the femur, placing the ACL under increased strain [130, 147, 151]. As shown in Figure 10, patients with ACL deficiency (green line) exhibit a markedly altered KIRM profile. Particularly, they demonstrate a significant reduction in internal rotation moment during the terminal stance phase [50], where healthy individuals typically generate a clear positive peak. A possible explanation is that ACL‐deficient patients adopt protective neuromuscular strategies to reduce rotational stress on the knee. As suggested by Fuentes et al. [50], this could involve increased hamstring activation or greater muscle co‐contraction during stance, both of which contribute to limiting internal tibial rotation and stabilising the joint in the absence of a functional ACL. Clinically, may serve as functional biomarkers to guide rehabilitation progression, tailor return‐to‐sport protocols and potentially identify patients at risk of graft failure.

Regarding the *COP* it is important to note that although it is derived from force data, COP is not a ‘pure kinetic’ variable: it describes a positional trajectory over time, making it a hybrid between kinetic and kinematic domains. This unique dual nature allows COP to reflect both external loading conditions and neuromuscular strategies used to maintain dynamic balance [126]. Changes in COP trajectory, amplitude, velocity, or complexity provide valuable insights into the strategies adopted by patients to maintain balance in the presence of musculoskeletal impairment [121, 126].

**Figure 11:**
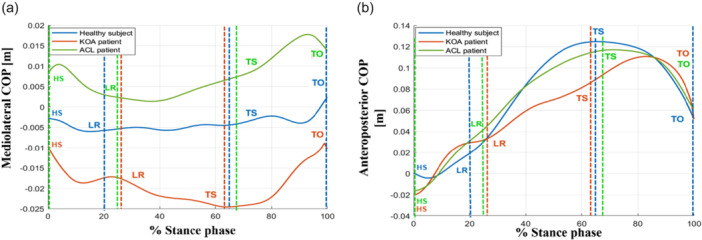
Mean mediolateral (a) and anteroposterior (b) centre of pressure during the gait cycle for healthy adults (blue), patients with KOA (red) and patients with ACL deficiency (green). Data represent the mean of five gait trials per subject, normalised to the gait cycle (%). ACL, anterior cruciate ligament; HS, heel strike; KOA, knee osteoarthritis; LR, loading response; TO, toe off; TS, terminal stance.

In individuals with KOA, COP is often observed to be laterally shifted during walking, particularly in the early‐ and mid‐stance phases, reflecting a strategy to offload the knee medial compartment [87, 105]. Furthermore, patients with KOA display increased mediolateral sway and greater variability in COP pattern, especially under challenging postural conditions [62, 138]. These findings are visually supported by Figure 11a, where the KOA patients (red line) demonstrate a marked lateral deviation of the mediolateral COP trajectory. As previously said, this displacement may reflect a compensatory shift in the base of support to redistribute joint loading away from the degenerated compartment [87, 105].

In the antero‐posterior (AP) direction, KOA patients (red line) are characterised by a COP pattern shifted anteriorly, especially during the first phase of gait [105] (Figure 11b). This shift could reflect altered timing in weight acceptance and push‐off, possibly due to pain avoidance, quadriceps weakness or proprioceptive deficits [121, 126].

Additionally, patients with ACL lesions (green line) tend to adopt more constrained and conservative postural strategies. This is reflected in a smoother and more regular mediolateral COP trajectory [110], with reduced lateral excursions across the stance phase (Figure 11a). A similar smooth and regular COP pattern [110] is also clear in the AP direction (Figure 11b), likely reflecting a more controlled and less dynamic weight shift strategy adopted to compensate for reduced knee stability. Consistently, previous studies have shown that COP velocity is lower in ACL patients compared to healthy controls [114]. This is qualitatively supported by Figure 11b, where the ACL patients display a flatter trajectory, suggesting a slower forward progression throughout stance phase.

From a clinical perspective, COP measures constitute a quantifiable, noninvasive and sensitive approach for evaluating dynamic postural control and knee joint loading strategies. COP has been extensively used to assess postural stability, detect neuromuscular compensation and track intervention outcomes in both static and dynamic tasks [121, 126].

## SAME DATA, DIFFERENT RESULTS: THE NEED FOR STANDARDISED PROTOCOLS

Gait analysis is becoming increasingly relevant in clinical decision‐making, offering valuable insights into lower limb biomechanics and guiding treatment planning and evaluation in musculoskeletal and neurological diseases [10, 20, 24, 42, 43, 60, 67, 74, 102, 145, 147]. However, while clinicians are encouraged to integrate gait metrics into practice, a critical aspect must be highlighted: the lack of protocol standardisation undermines data comparability and interpretation across centres and systems [6, 44, 63, 97].

A protocol defines not only the marker‐set (i.e., the type, number and position of reflective markers), but also the biomechanical model, including assumptions about segment definition, joint degrees of freedom, rotation sequences and reference frames. It also encompasses data collection, processing and reporting procedures [44]. These methodological details, often overlooked in routine clinical reports, have a significant impact on the reliability and interpretability of gait outcomes. Current protocols vary substantially, not only in the marker placement and anatomical references, but also in the kinematic and kinetic variables derived, as well as in terminology and conventions [44, 97]. Numerous studies have demonstrated that even minor methodological differences, such as marker placement, segment definitions, rotation sequences and filtering parameters, can result in clinically meaningful variations in output data [15, 29, 38, 44, 79, 98, 103, 156]. Regarding kinematic outcomes, discrepancies are particularly evident in the frontal and transverse planes, which are more susceptible to soft tissue artefacts and model‐related assumptions [44, 79, 98, 103]. Furthermore, differences in data filtering methods have been shown to strongly affect kinetic outputs. This is particularly relevant when computing joint moments via inverse dynamics, as these calculations involve derivatives of position data and are therefore highly sensitive to the chosen filtering strategy. In this context, even minor variations in data processing can significantly impact joint moment estimation. For example, a change of only 0.5 Hz in the cut‐off frequency of the filtering process has been shown to produce larger errors than those introduced by variations in body segment inertial parameters [18, 109]. Notably, methodological adjustments such as modifying marker weighting schemes or filter settings during data reconstruction can lead to errors in knee joint moments computation of up to 13% [115]. As observed for kinematic variables, also kinetic parameters are most affected in the frontal and transverse planes [18] further emphasising the tight interdependence between the two domains.

Another critical issue relates to the lack of training and standardisation among healthcare professionals involved in instrumented gait analysis. Although physiotherapists, occupational therapists and orthopaedic surgeons may all participate in gait assessment, no unified training framework or standardised clinical protocol currently exists. This heterogeneity in professional expertise introduces further variability in data interpretation and clinical decision‐making [63].

This absence of internationally recognised clinical standards represents a broader challenge for the field, as standardisation is essential to ensure safety, effectiveness and reproducibility across healthcare systems, enabling evidence‐based clinical decisions and supporting integration within national healthcare frameworks and medical pathways [6, 44, 63, 97].

A further step towards improving the clinical integration of gait analysis involves addressing not only methodological heterogeneity, but also the lack of consistent terminology across studies. This issue is not limited to biomechanical variables alone, but extends to rehabilitation protocols, where terms such as ‘accelerated rehabilitation’, ‘delayed weight‐bearing’ or ‘early ROM’ are frequently used without standardised definitions [120]. Such variability hinders cross‐study comparability and weakens the translational value of gait‐derived parameters. Future initiatives aimed at consolidating terminology frameworks would therefore represent an essential advancement in support of evidence‐based, interoperable clinical applications. In addition to improving standardisation, further investigations should focus on conducting prospective comparative studies to validate the clinical value of gait analysis across different rehabilitation settings and musculoskeletal conditions. Such works could help define reference thresholds for pathological gait patterns, monitor treatment response over time and support the integration of motion analysis into personalised care pathways.

In conclusion, while this review aims to provide clinicians with a practical framework for interpreting gait data, it is crucial to acknowledge the methodological heterogeneity underlying current practices. There is a pressing need for consensus‐driven protocols that balance biomechanical rigor with clinical feasibility. The establishment of shared standards will be key to advancing gait analysis as a robust, reliable, and clinically impactful tool in musculoskeletal care.

## AUTHOR CONTRIBUTIONS

All authors contributed to the conception and design of the study. All authors read, reviewed and approved the final version of the manuscript. Giovanni Spallone and Letizia Mancini are the first authors with equal contributions.

## CONFLICT OF INTEREST STATEMENT

The authors declare no conflicts of interest.

## ETHICS STATEMENT

The authors have nothing to report.

## Data Availability

The authors have nothing to report.
